# The regulation of lncRNAs and miRNAs in SARS-CoV-2 infection

**DOI:** 10.3389/fcell.2023.1229393

**Published:** 2023-07-27

**Authors:** Yuhao Lin, Qiqi Sun, Bao Zhang, Wei Zhao, Chenguang Shen

**Affiliations:** BSL-3 Laboratory (Guangdong), Guangdong Provincial Key Laboratory of Tropical Disease Research, School of Public Health, Department of Laboratory Medicine, Zhujiang Hospital, Southern Medical University, Guangzhou, China

**Keywords:** SARS-CoV-2, COVID-19, non-coding RNA, miRNA, lncRNA, mechanism

## Abstract

The coronavirus disease 2019 (COVID-19) was a global endemic that continues to cause a large number of severe illnesses and fatalities. There is increasing evidence that non-coding RNAs (ncRNAs) are crucial regulators of viral infection and antiviral immune response and the role of non-coding RNAs in SARS-CoV-2 infection has now become the focus of scholarly inquiry. After SARS-CoV-2 infection, some ncRNAs’ expression levels are regulated to indirectly control the expression of antiviral genes and viral gene replication. However, some other ncRNAs are hijacked by SARS-CoV-2 in order to help the virus evade the immune system by suppressing the expression of type I interferon (IFN-1) and controlling cytokine levels. In this review, we summarize the recent findings of long non-coding RNAs (lncRNAs) and microRNAs (miRNAs) among non-coding RNAs in SARS-CoV-2 infection and antiviral response, discuss the potential mechanisms of actions, and prospects for the detection, treatment, prevention and future directions of SARS-CoV-2 infection research.

## 1 Introduction

Coronavirus disease 2019 (COVID-19), a respiratory infectious disease caused by the severe acute respiratory syndrome coronavirus 2 (SARS-CoV-2), had been first reported in December 2019 and caused a worldwide pandemic. Because of its high transmissibility and fatality, the World Health Organization (WHO) declared a public health emergency of international concern on 30 January 2020. As of 18 June 2023, over 768 million confirmed cases and over 6.9 million deaths have been reported globally (World Health Organization, 2020). The clinical manifestation of SARS-CoV-2 infection range from asymptomatic infection to respiratory symptoms of high fever and dry cough, and in severe cases to pneumonia, which may progress to fatal acute respiratory distress syndrome (ARDS) and multiple organ dysfunction syndrome (MOD). The secretion of various cytokines during infection is closely related to clinical manifestations and cytokine storm is closely associated with ARDS and MOD (Sun et al., 2020). The breakthrough infection rate for SARS-CoV-2 has increased since the Omicron variant became mainstream (Wang X. et al., 2022). Therefore, we must advance our understanding of the infection mechanisms of SARS-CoV-2 and develop a new generation of therapeutic medicines and vaccines. Since ncRNAs and viruses attracted the attention of so many researchers, it has been confirmed that ncRNAs contribute significantly to host-virus interactions (Damas et al., 2019). The infection of SARS-CoV-2 may also cause ncRNA dysregulation, which in turn leads to various impacts on the host body and results in the emergence of immune evasion response, inflammatory response, and antiviral response.

Although about two-thirds of the human genome is actively transcribed, only about 1.9% of the genome encodes proteins (Mattick, 2001; Consortium, 2004; Djebali et al., 2012). NcRNAs are a large class of RNA transcripts that are transcribed from the genome but lack the ability to encode proteins (Mohapatra et al., 2021). NcRNAs are classified into different categories based on their length, shape, and function. Regulatory ncRNAs are mainly divided into four categories, including microRNAs (miRNAs), long non-coding RNAs (lncRNAs), circular RNAs (circRNAs) and piwi-interacting RNAs (piRNAs) (Zhao et al., 2020; Lou et al., 2021). SARS-CoV-2 infection can significantly alter the expression profile of host ncRNAs, and some affected ncRNAs have been confirmed to play crucial roles in viral replication and/or host immune responses. Recent studies suggest that ncRNAs, especially microRNAs (miRNAs), long non-coding RNAs (lncRNAs) and circular RNAs (circRNAs), may play an important role during SARS-CoV-2 infection. There are relatively few research on how SARS-CoV-2 regulate the level of ncRNAs in host cells because the field of ncRNAs-SARS-CoV-2 is still in its early stages. The generally accept theory holds that SARS-CoV-2 RNA identification by the host pathogen recognition receptor promotes the body’s own innate immune response and IFN can activate certain lncRNAs. For example, the expression of NRIR/lncCMPK2 can be activated by IFN and then modulated by signal transducer and activator of transcription 2 (STAT2) (Ding et al., 2022). SARS-CoV-2-derived miRNAs can alter interferon, WNT, and mTOR signaling as well as autophagy (Khan et al., 2020). Zhang et al. discovered that LINE-1 or co-infected retrovirus (HIV) may retrotranscribe and integrate SARS-CoV-2 RNA into the human genome (Zhang et al., 2020). We hypothesize that SARS-CoV-2 may integrate into the host cell genome and suppress the expression level of ncRNAs similarly to how HBV dose (Qiu et al., 2018). In this paper, we will focus on how lncRNA and miRNA affect the process of SARS-CoV-2 infection, their possible regulatory mechanisms, and their application prospects and research directions.

## 2 Possible regulation mechanisms of lncRNAs in SARS-CoV-2 infection

**FIGURE 1:**
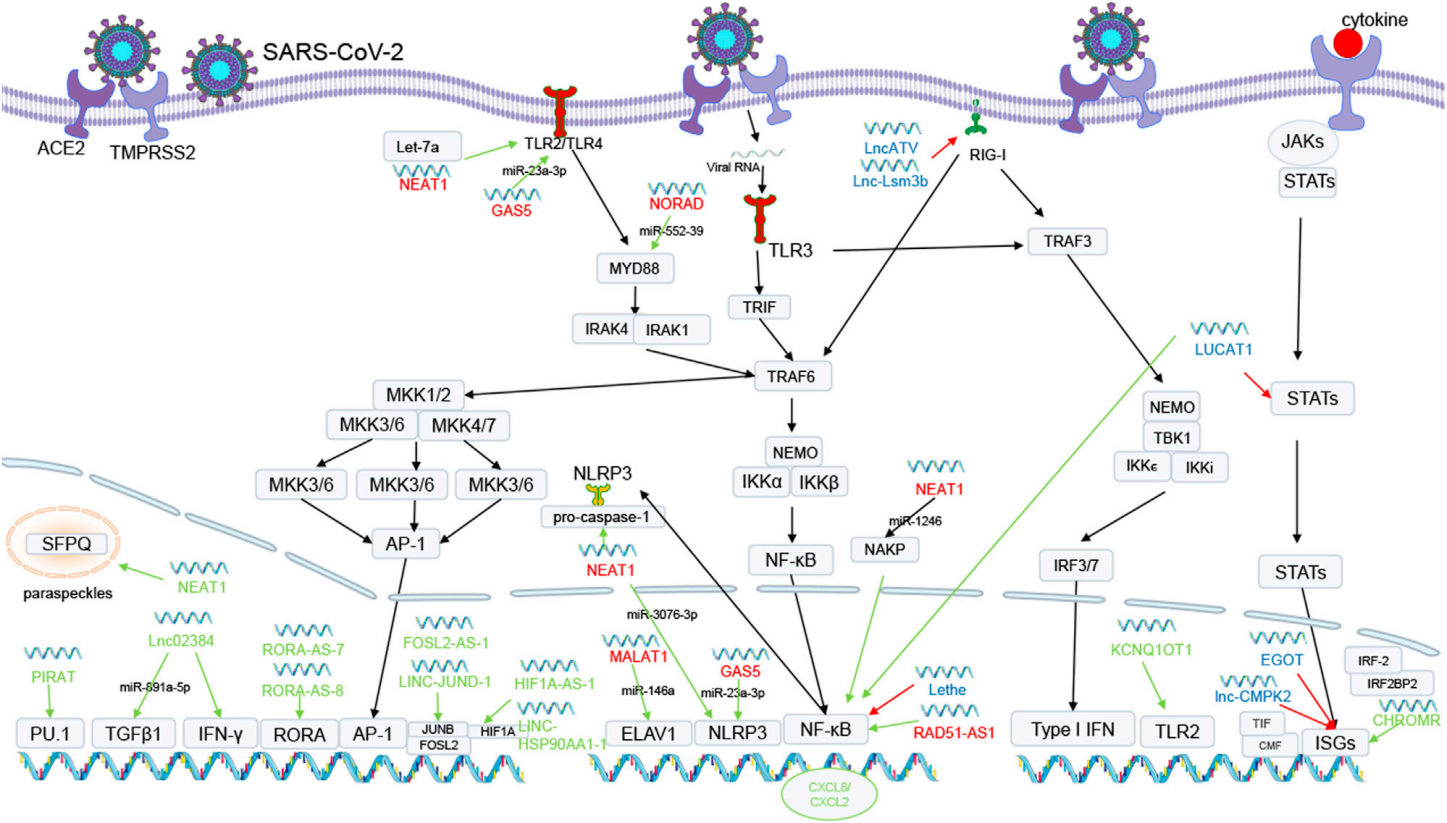
The possible mechanisms of lncRNAs in SARS-CoV-2 infection. Green lines mean upregulating expression or promoting process. Red lines mean downregulating gene expression. Green lncRNAs is antiviral response related lncRNAs. Blue lncRNAs is immune escape related lncRNAs. Red lncRNAs is cytokine storm related lncRNAs. Some lncRNAs boost antiviral responses by triggering innate or adaptive immunity or by increasing the production of antiviral components. NEAT1 relocated splicing factor proline- and glutamine-rich protein (SFPQ) to the paraspeckles, removing the transcriptional inhibitory effects of SFPQ. PIRAT interacts with PU.1 to produce alarmin S100A8/A9. Lnc02384 enhances the expression of IFN-IFN-γ-encoding genes and increases TGFβ-1 mRNA transcript levels by uptaking miR-891a-5p. RORA-AS-7 and RORA-AS-8 regulate RORA genes, LINC-JUND-1 and FOSL2-AS-1 regulate FOSL2 and JUNB, and HIF1A-AS-1 and LINC-HSP90AA1-1 regulate HIF1A, all of which are associated with AP-1 and promote T-cell activation, differentiation and enhancing effects. KCNQ1OT1 interacts with the TLR2 gene which identifies PAMP and functions through MYD88 and TRAF6 to cause NF-κB activation. CHROMR isolated nuclear transcriptional co-blocker IRF2BP2 and IRF-2. Other lncRNAs will aid SARS-CoV-2 in escaping the immune system and escalating bodily tissue harm. LncATV and Lnc-Lsm3b bind and inhibit RIG-I-mediated innate immune activation. LncRNA Lethe binds to NF-κB to inhibit Rela DNA binding and target gene activation. LncRNA LUCAT1 limits the expression of STAT targets and reduces the generation of interferon. LncRNA EGOT and CMPK2 are involved in the control of ISGs. NEAT1 enhances caspase-1 activation by attaching to pro-caspase-1 and encouraging the activation of NLRP3 inflammasomes. NEAT1 as well as competitively binds miR-1246 and releases NAKP, which mediates NF-κB activation. Additionally, upregulated NEAT1 competitively binds Let-7a and releases TLR4 from it, activating TLR4 and stimulating downstream signaling. GAS5 decreases miR-223-3p expression, which increases NLRP3 expression levels and promotes pyroptosis and downregulates miR-23a-3p levels, which can inhibit the TLR4/TNF-α/IL-10 signaling pathway. MALAT1 suppresses the creation of NLRP3 inflammasomes by sponging miR-23C, preventing its repressive impact on the target gene ELAV1. NORAD is involved in the NF-κB signaling pathway through the miR-552-39/MYD88 axis in TNF-α induced apoptosis and inflammation. RAD51-AS1 leads to the expression of pro-inflammatory cytokines.

Reviewing a number of viral infections, it has been concluded that they may alter the host’s lncRNA expression and control the infection process through various regulatory mechanisms (Liu et al., 2019). Similarly, various lncRNAs are engaged in the SARS-CoV-2 infection process, and several studies like bio conductivity analyses have demonstrated that lncRNAs are associated with the infection and pathology of SARS-CoV-2. In BALF and PMBC samples, Moazzam-Jazi et al. discovered 207 and 223 substantially changed lncRNAs; some of them were connected to immune-related genes strongly linked to possible transcriptional augmentation and transcriptional repression (Moazzam‐Jazi et al., 2021). In comparison to control NBHE cells, Vishnubalaji et al. found that 155 lncRNAs were upregulated and 195 were downregulated in response to SARS-CoV-2 infection (Vishnubalaji et al., 2020). In vaccination breakthrough (VBT) patients and unvaccinated COVID-19, Chattopadhyay et al. discovered 722 differently expressed lncRNAs (153 upregulated and 574 downregulated) (Chattopadhyay et al., 2022). By analyzing RNA-seq data, Turjya et al. discovered 21 lncRNAs that were differentially expressed, of which 9 were upregulated and 12 were downregulated (Turjya et al., 2020). We assembled a list of lncRNAs and their functions in SARS-CoV-2 infection (as shown in Table 1). To aid readers in understanding the mechanism, we have also provided a figure (as shown in Figure 1). We categorized the possible mechanisms of lncRNAs’ involvement in SARS-CoV-2 infection into three categories, which are detailed below: immune evasion-related, cytokine storm-related, and antiviral response-related mechanisms.

**TABLE 1:** Function and related target of lncRNAs in SARS-CoV-2 infection.

Related target	lncRNA	Initial function	Immune response	Reference
IFN	lncRNA-CMPK2	Blocking the transcription of ISGs	Limit innate immune	Enguita et al. (2022)
	EGOT	Downregulate the expression of several ISGs	Limit innate immune	Mukherjee et al. (2021)
	LUCAT1	Reduce the expression of STAT-targets CXCR4 and NAMPT	Limit innate immune	Aznaourova et al. (2022)
	CHROMR	Promote the transcription of ISGs	Promote innate immune	van Solingen et al. (2022)
	lnc02384	Increase the production of IFN-γ	Promote innate immune	Moazzam‐Jazi et al. (2021)
	NETA1	Promote the expression of DDX60 and RIG-I	Promote innate immune	Ma et al. (2017)
TNF-α/IL-10	GAS5	Induced NLRP3、IL-10、TNF-α	Inflammation	Morenikeji et al. (2021)
NF-κB	LUCAT1	Induced expression of proinflammatory markers CXCL2 and CXCL8	Inflammation	Aznaourova et al. (2022)
	PIRAT	Reduce alarmin production	Inflammation	Aznaourova et al. (2022)
	KCNQ1OT1	Interact with upregulated TLR2	Inflammation	Turjya et al. (2020)
	NORAD	Induced IL-6、IL-10、CSF3、CXCL10、TNF-α	Inflammation	Morenikeji et al. (2021)
	RAD51-AS1	Induced IL-6、TNF-α、CCL2	Inflammation	Morenikeji et al. (2021)
	MALAT1	Induced TNF-α、IL-6、IL-1 and CCL2	Inflammation	Moazzam‐Jazi et al. (2021)
	NEAT1	Induced TNF-α, IL-6, and IL-1, NLRP3, as well as CCl2, Ccl3, and CXCL10	Inflammation	Moazzam‐Jazi et al. (2021)
Th17 cell differentiation	CCR7-AS-1, LEF1-AS-1	T cell activation and differentiation	Adaptive immune	Zheng et al. (2020)
	LINC-CCR7-2			
	LINC-TCF7-1			
	TCF7-AS -1			
MAPK	LINC-JUND-1, LINC-HSP90AA1-1, HIF1A-AS-	T cell activation and differentiation	Adaptive immune	Zheng et al. (2020)

Few studies have investigated the direct binding of lncRNAs to the SARS-CoV-2 genome and their effects on the virus’ replication process because, in contrast to miRNAs, lncRNAs often contain more complicated secondary structures. However, MALAT1 which is considered to serve a significant regulatory function in SARS-CoV-2 infection is the promoter of HIV-1 transcription and encourages HIV-1 infection (Qu et al., 2019). The SARS-CoV-2 genome or Spike mRNA may interact with lncRNA H19, FENDRR, HOTAIR, LINC01505, HOTAIRM1, PVT1, and AL392172.1 to control SARS-CoV-2 replication (Moazzam-Jazi et al., 2021; Natarelli et al., 2021), according to another studies. The above information points to a possible role for lncRNAs in binding to and controlling the SARS-CoV-2 genome, while further study is required in this area.

### 2.1 Antiviral response

#### 2.1.1 lncRNAs regulate innate immunity

By triggering type I interferon-dependent antiviral responses and the start of inflammatory responses, innate immunity acts as the first line of defense against invasive viruses (Chiale et al., 2022). Some lncRNAs serve a function in enhancing innate immune initiation in addition to the traditional innate immune initiation pathway. In the infection of SARS-CoV-2, lnc02384 can act as a possible transcriptional enhancer of neighboring IFN-γ-encoding genes (Moazzam-Jazi et al., 2021) and promote IFN-γ synthesis; in addition, in the study on melanoma lnc02384 was discovered to uptake miR-891a-5p (Zhang et al., 2021) and decreased levels of miR-891a-5p were able to increase TGFβ-1 mRNA transcript levels (Shen et al., 2023), which ultimately has the effect of promoting the expression and activation of other cytokines including interferon-γ (IFN-γ) and tumor necrosis factor-α (TNF-α). Downregulating PIRAT weakens its ability to prevent PU.1 from producing alarmin S100A8/A9, whereas upregulating LUCAT1 increases alarmin expression in addition to its capacity to promote the expression of the pro-inflammatory markers CXCL2 and CXCL8 at the expense of JAK/STAT pathway targets (Aznaourova et al., 2022). The expression of NEAT1 is significantly upregulated in SARS-CoV-2 infection, and its antiviral response mechanisms may be referenced to its mechanisms in hantavirus infection, where NEAT1 relocated splicing factor proline- and glutamine-rich protein (SFPQ) from the potential promoter regions of several antiviral genes to the paraspeckles, removing the transcriptional inhibitory effects of SFPQ. This phenomenon would facilitate the expression of DDX60 and retinoic acid-inducible gene I (RIG-I), aiding the IFN response and preventing viral infection (Ma et al., 2017). CHROME increased in patients with SARS-CoV-2 infection. The nuclear transcriptional co-blockers IRF2BP2 and IRF-2, which have a repressive transcriptional effect on IFN-stimulated genes (ISGs), are isolated by CHROMR, which in turn causes ISGs to be released to aid the antiviral response (van Solingen et al., 2022). Downregulated KCNQ1OT1 interacts with upregulated TLR2, which identifies PAMP and functions through MYD88 and TRAF6 to cause nuclear factor kappa-light-chain-enhancer of activated B cells (NF-κB) activation, cytokine production, and inflammatory responses (Turjya et al., 2020), and their connection may aid in the elimination of invasive viruses.

#### 2.1.2 lncRNAs regulate adaptive immunity

T cells also play a critical role in defending viruses: CD4^+^ T cells help B cells produce antibodies and orchestrate the immune system’s response, whilst CD8^+^ T cells destroy infected cells to inhibit viral replication (Vabret et al., 2020). Some lncRNAs promote T cells activation and differentiation and even enhance T cell functions, such as LINC-JUND-1, FOSL2-AS-1 that regulate FOSL2 and JUNB, paralogs of AP-1; and HIF1A-AS-1, LINC-HSP90AA1-1 that regulate HIF1A that interacts with JUN genes and enhances T cell functions to promote viral elimination; RORA-AS-7, RORA-AS-8 regulate RORA genes together with AP-1 to regulate T cell differentiation. CCR7-AS-1, LEF1-AS-1, LINC-CCR7-2, LINC-TCF7-1, and TCF7-AS-1 are associated with T cell activation and differentiation genes related (Zheng et al., 2020). Although the current study by Agirre et al. showed that lncRNAs are specifically expressed in each phase of the humoral immune response (Agirre et al., 2019), a complete and comprehensive understanding of the specific response mechanisms of lncRNAs in the humoral immune response is lacking. Further in-depth research in this area may assist people in creating more effective and cutting-edge antiviral drugs and vaccines against a variety of highly pathogenic and emerging infectious diseases.

### 2.2 Immune evasion

While SARS-CoV-2 may resist viral RNA identification, evade retinoic acid-inducible I-like receptor (RLR) signaling, and decrease IFN production through its own proteins such as NSP5, NSP6, NSP7, and ORF9b (Beyer and Forero, 2022), IFN plays a crucial role in the antiviral response. Additionally, viruses like influenza frequently make use of lncRNAs that do not require interferon activation to boost their reproduction through several molecular pathways involved in the integrity of the viral genome and replication machinery. Due to the strong resemblance between SARS-CoV and SARS-CoV-2, it has been demonstrated that the influenza virus and SARS-CoV exhibit comparable expression patterns in the lung tissue of infected mice (Hartshorn, 2020). We hazard a guess that SARS-CoV-2 may also hijack lncRNAs to control ISGs, RIG-I, JAK-STAT, NF-κB, and other pathways to increase replication and infection.

The expression of lncRNA-CMPK2, which is involved in the control of ISGs, is elevated in SARS-CoV-2 infection. With ISGs, lncRNA-CMPK2 aids in directing transcriptional repressors or chromatin-modifying factors to sites in a subset of ISGs. This results in a block to transcription or the induction of a repressive chromatin state in these regions, downregulating the IFN response (Kambara et al., 2014). In addition, in SARS-CoV-2 infected cells, through the utilization of NF-κB, RIG-I detection of viruses causes an increase in EGOT expression levels, which in turn causes the expression of several ISGs (GBP1, ISG15, Mx1, BST2, ISG56, IFI6 and IFITM1) to be downregulated (Carnero et al., 2016).

Although their precise mechanisms are unknown, lncRNAs such as DANCR, TUG1, and Gm26917 are engaged in the control of the RIG-I pathway. It is possible that the process of LncATV and Lnc-Lsm3b might serve as a guide for the investigation of these lncRNAs’ mechanisms. For instance, LncATV binds and inhibits RIG-I-mediated innate immune activation (Fan et al., 2019). Lsm3b’s GA-rich sequence produces a double-stranded RNA stem-loop structure that competitively binds to the C-terminal domain of RIG-I, stabilizing the contact between the RIG-I caspase recruitment domain (CARD) and helicase domain, keeping it in an auto-repressed conformation (Bird, 2018). In addition, RIG-I is kept in an inactive state by Lsm3b, which also effectively suppressed the dsRNA-induced ATPase activity of RIG-I and disturbed TRIM25-mediated K63-linked ubiquitination of RIG-I upon viral infection (Jiang et al., 2018).

The human interferon responses are also thought to be negatively regulated by the lncRNA LUCAT1 (Agarwal et al., 2020). Due to its major effect on the Janus kinase/signal transducers and activators of transcription (JAK-STAT) pathway, it can limit the expression of STAT targets, reduce the generation of interferon, and eventually encourage the infection and replication of the SARS-CoV-2 virus.

On the other hand, lncRNA Lethe induced by inflammatory factors TNF-α and IL-1β binds to NF-κB to inhibit Rela DNA binding and target gene activation (Rapicavoli et al., 2013), reducing the release of inflammatory factors with anti-infective effects may be the potential mechanisms of lncRNA-assisted SARS-CoV-2 immune evasion.

The study of lncRNAs in SARS-CoV-2 immune evasion is lacking, and its mechanisms of innate immune evasion are primarily extrapolated from other viruses. While the mechanisms of lncRNA in humoral immunity are less well understood, Only Agirre et al. demonstrated that lncRNAs regulate essential genes during B-cell immune activation (Agirre et al., 2019). There is no evidence that lncRNAs play a role in the humoral immune evasion mechanisms of SARS-CoV-2.

### 2.3 Cytokine storm

Type II alveolar epithelial cells that have been infected by SARS-CoV-2 undergo apoptosis and cytokinesis, which kills the cells. Additionally, it activates lung macrophages and causes the release of pro-inflammatory cytokines and chemokines, which tends to the M1 phenotype (Morris et al., 2020). In conclusion, SARS-CoV-2 infection triggers the inflammatory cascade response in humans, and the ensuing unchecked inflammatory factors finally result in a cytokine storm, which has devastating outcomes including ARDS and systemic multi-organ failure.

22 lncRNAs with the ability to target the 10 cytokines overexpressed in the COVID-19 cytokine storm were found by Morenikeji et al., eight of which target two or more cytokines (Morenikeji et al., 2021). For instance, NORAD specifically targets five crucial cytokines (IL-6, IL-10, CSF3, CXCL10, and TNF-α), and its possible mechanisms may be the involvement of the NF-κB signaling pathway through the miR-552-39/MYD88 axis in TNF-α induced apoptosis, inflammatory, and oxidative stress (Lei et al., 2022), which in turn leads to an increase in multiple cytokines. RAD51-AS1 targets three significant cytokines (IL-6, TNF-α, CCL2), and its possible mechanisms may express in response to cellular damage from viral replication within macrophages in SARS-CoV-2 infection, leading to the expression of pro-inflammatory cytokines (Morenikeji et al., 2021). In addition, RAD51-AS1 was discovered to be associated with the NF-κB pathway in inflammatory bowel disease (Papoutsopoulou and Campbell, 2021), which could account for the upregulation expression of IL-6, TNF-α, and CCL2. GAS5 targets two crucial cytokines (IL-10 and TNF-α), and the possible mechanisms may be that upregulating GAS5 expression decreases miR-223-3p expression, which increases NLRP3 expression levels and promotes pyroptosis (Mo et al., 2022). GAS5 also downregulates miR-23a-3p levels, which can inhibit the release of inflammatory substrates by inhibiting the TLR4/TNF-α/IL-10 signaling pathway (Gao and Huang, 2021).

NEAT1 and MALAT1 target more than 95% of the genes and proteins among the many differential expression lncRNAs (Laha et al., 2021) and are essential for the development of cytokine storms. By attaching to pro-caspase-1 and encouraging the activation of NLRP3, NLRC4, and AIM2 inflammasomes, NEAT1 enhances caspase-1 activation, cytokine production, and pyroptosis (Zhang P. et al., 2019). Through the absorption of miR-3076-3p and reduction of the latter function to control the expression of NLRP3 (Zhang M. et al., 2019). Additionally, upregulated NEAT1 competitively binds Let-7a and releases TLR4 from it, activating TLR4 and stimulating downstream signaling (Zhang and Niu, 2019). NEAT1 as well as competitively binds miR-1246 and releases NAKP, which mediates TNF-α and IL-1 induced NF-κB activation and severe inflammatory response, causing a serious inflammatory reaction (Bai et al., 2018). MALAT1 also suppresses the creation of NLRP3 inflammasomes by sponging miR-23C, preventing its repressive impact on the target gene ELAV1, which is the upstream gene of the NLRP3 inflammasome (Li et al., 2017). Additionally, MALAT1 functions as a molecular sponge for miR-146a, lowering miR-146a levels to lessen its inhibition of the generation of inflammatory responses induced by LPS (Dai et al., 2018). In addition to interacting with miRNAs, MALAT1 also binds to E2H2, which in ER-negative breast cancer works as a coactivator of RelA and RelB and upregulates the production of NF-κB targeted genes including IL-6 (Lee et al., 2011). In addition to the aforementioned, it can stimulate neutrophil chemotaxis by controlling p300-dependent IL-8 (Wei et al., 2019).

## 3 Possible regulation mechanisms of miRNAs in SARS-CoV-2 infection

Lots of studies have confirmed that miRNAs also have an important impact on SARS-CoV-2 infection, which causes dysregulation of host miRNA expression and affects the infection process through various interactions. Chow and Salmena’s study identified 128 human miRNAs with the potential to target the SARS-CoV-2 genome. Their results showed that six of these 128 miRNAs were differentially expressed. Four of them were downregulated (let-7a-3p, miR-135b-5p, miR-16-2-3p, and miR1275), and two were upregulated (miR-155-3p and miR-139-5p) (Chow and Salmena, 2020). Li et al. found that 73 miRNAs were differentially expressed in the peripheral blood of SARS-CoV-2 patients compared with healthy individuals, of which 35 were upregulated and 38 were downregulated. Among these miRNAs, miR-16-2-3p, miR-6501-5p, and miR-618, their expression change was higher than 1.5-fold compared with the control group (Li C. et al., 2020). The study by Wang et al. found that SARS-CoV-2 patients had downregulated overall miRNA expression levels, including 41 miRNAs (Wang Y. et al., 2022). The study by Fernández-Pato et al. detected a total of 200 miRNA expression changes in SARS-CoV-2 patients, of which 142 miRNAs levels were upregulated and 58 were downregulated (Fernández-Pato et al., 2022). Another study demonstrated differential miRNA expression in SARS-CoV-2 patients of different severity. MiR-146a-5p, miR-21-5p, and miR-142-3p were consistently downregulated, and miR-3605-3p was consistently upregulated; miR-15b-5p, miR-486-3p, and miR-486-5p were only upregulated in severe SARS-CoV-2 patients; miR-181a-2-3p, miR-31-5p, and miR-99a-5p were only downregulated in severe patients (Tang et al., 2020). Wilson’s study found that miR-4454 + 7975, miR-1285-5p, miR-320e, and miR-29b-3p were upregulated, while miR-451a and miR-144-3p were downregulated (Wilson et al., 2022).

**FIGURE 2:**
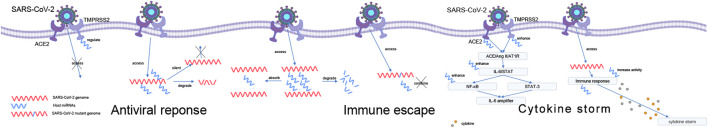
Possible role of miRNAs during SARS-CoV-2 infection. Antiviral response: regulates the expression of ACE2 and TMPRSS2 and direct binding to the viral genome. Immune evasion: interfering with host miRNA and binding site mutations. The way of miRNA works during the cytokine storm caused by SARS-CoV-2: IL-6 amplifies activation pathway and causes cytokine storm by regulating anti-inflammatory cytokine genes.

We have preliminarily sorted out the binding sites and functions of some miRNAs during SARS-CoV-2 infection (as shown in Table 2). To aid readers in understanding the mechanism, we have also provided a figure (as shown in Figure 2). We preliminarily divide the potential mechanisms of miRNAs in SARS-CoV-2 infection into antiviral response related, immune escape related, and cytokine storm related, and explain them one by one below.

**TABLE 2:** The role of some host miRNAs in SARS-CoV-2 infection.

miRNA	Binding sites	Function	Reference
miR-200 family (↑)	ACE2	proinflammatory as well as anti-inflammatory	Lu et al. (2020), Pierce et al. (2020), Chauhan et al. (2021), Meidert et al. (2021), Pimenta et al. (2021)
		virus entry	
miR-421(↓)	ACE2	Aggravating inflammatory response	Hum et al. (2021)
miR-155(↑)	ACE2	Reducing proinflammatory cytokines	Chow and Salmena (2020), Garg et al. (2021), Wyler et al. (2021)
	IL-1	Increased antiviral responses	
miR-483-3p(↑)	ACE2	ACE2 expression downregulated	Kemp et al. (2014)
miR-125a-5p(↓)	ACE2	Increased ACE2 protein expression level	Nersisyan et al. (2020a)
let-7b-5p、	TMPRSS2	TMPRSS2 expression downregulated	Nersisyan et al. (2020a), Chow and Salmena (2020), Mukhopadhyay and Mussa (2020), Wang et al. (2022b)
let-7a-5p、let-7d-5p(↓)	IL6/IL6R	Related to inflammatory responses	
miR-21(↑)	SARS-CoV-2 genome	Regulating inflammatory response	Sheedy (2015), Nersisyan et al. (2020a), Nersisyan et al. (2020b), Fulzele et al. (2020), Haddad and Al-Zyoud (2020), Srivastava et al. (2020), Garg et al. (2021)
		27 binding sites on SARS-CoV-2 genome	
miR-125a-3p(↑)	SARS-CoV-2 gene S	Inhibits the cleavage of the S gene	Chow and Salmena (2020), Demirci and Adan (2020), Fulzele et al. (2020), Khan et al. (2020)
miR-1307-3p(↓)	SARS-CoV-2 3′UTR	Promoting inflammatory responses	Arisan et al. (2020), Balmeh et al. (2020)
		Involved in TGF-β signaling	
miR- 5047	SARS-CoV-2 multiple	Inhibits cleavage of the ORF1a/b polyprotein gene	Fulzele et al. (2020), Pierce et al. (2020)
miR-323a-5p(↑)	SARS-CoV-2 ORF1a/b	antiviral infection	Khan et al. (2020)
miR-24	SARS-CoV-2 furin	Regulation of furin and TGF-β1 content	Hum et al. (2021)
miR-16	SARS-CoV-2 genome	apoptosis	Hum et al. (2021)
	BCL2		
miR-146a(↓)	IL-1	Inflammation	Rakhmetullina et al. (2020)
	IL-6 TNFα		
miR-1207-5p		Inflammation	Bertolazzi et al. (2020)
miR-320 family(↑)	IL-8	Inflammation	Fernández-Pato et al. (2022)
	IL-6		
	IP10		

Note: ↓: downregulated; ↑: upregulated.

### 3.1 Antiviral response

MiRNAs may be an important antiviral tool (Trobaugh and Klimstra, 2017), which can regulate the innate and adaptive immune systems (Ambros, 2001; Trobaugh and Klimstra, 2017) and can also cause silencing or degradation of viral group genes by directly binding them. How miRNAs resist SARS-CoV-2 may be mainly of two types: regulation of ACE2 and TMPRSS2 expression and direct binding to the viral genome to silence or degrade it.

#### 3.1.1 Regulate the expression of ACE2 and TMPRSS2

During infection, SARS-CoV-2 interacts with cells through the spike protein, and angiotensin-converting enzyme 2 (ACE2) acts as a receptor to bind to the spike protein and mediate virus entry. The spike protein is cleaved and activated by the cell surface transmembrane protease serine 2 (TMPRSS2) to facilitate membrane fusion and entry (Hoffmann et al., 2020; Pierce et al., 2020). Inactivation of ACE2 or TMPRSS2 is an initial step that can inhibit host cell entry.

Many studies have shown that miRNAs can regulate the expression of ACE2. The study by Lu et al. showed that miR-200c may reduce the risk of infection by inhibiting ACE2 (Lu et al., 2020). Whereas miR-125b and miR-18 directly target the 3′-UTR of ACE2 mRNA (Widiasta et al., 2020). In a study using bioinformatics analysis by Nersisyan et al., it was described that the miR-125a, miR-141, let-7e, and miR-200 families were downregulated in SARS-Cov-2 infection, thereby regulating the expression of ACE2 (Nersisyan et al., 2020a). A bioinformatics study suggests that miR-302c-5p and miR-16-5p may affect SARS-CoV-2 infection by regulating the ACE2 receptor-associated network (Li et al., 2022). The study by Pierce et al. found three miRNAs targeting ACE2 mRNA, namely, miR-9-5p, miR-218-5p, and miR-483-3p. Differential expression of these three miRNAs also alters resistance to SARS-CoV-2 infection (Pierce et al., 2020). Several studies have shown that miR-421, miR-143, and miR-483-3p can reduce ACE2 protein expression levels (Kemp et al., 2014; Lambert et al., 2014; Mukhopadhyay and Mussa, 2020). Another study reported that miR-200b-3p, miR-200 c-3p, and miR-429 antagonise ACE2, and let-7c-5p, miR-98-5p, and miR-4500 regulate TMPRSS2 (Chauhan et al., 2021). In addition, the study by Kaur et al. found that miR-214, miR-98 and miR-32 could inhibit TMPRSS2, especially miR-32 (Kaur et al., 2021).

#### 3.1.2 Direct binding to the viral genome

MiRNAs can act as antivirals by binding to viral RNAs to inhibit their replication. A computer analysis study by Fulzele et al. found 873 common miRNAs targeting the SARS-CoV-2 genome (Fulzele et al., 2020). The bioinformatics study by Khan et al. found that most host miRNAs target the ORF1ab region, followed by the S region. In addition, the M, N, ORF3a, ORF7a, ORF8, 5′UTR, and 3′UTR regions were also targeted by host miRNAs. Three host miRNAs (miR-17-5p, miR-20b-5p, and miR-323a-5p), which target the viral genome, were shown to have significant antiviral effects during infection. This study also showed that miR-506-3p, miR-6817-5p, and miR-12199 could interact with the nucleocapsid (Khan et al., 2020). Another study identified six miRNAs (miR-21-3p, miR-195-5p, miR-16-5p, miR-3065-5p, miR-424-5p, and miR-421) that showed a high probability of binding coronavirus in all analyses of that study, with miR-21-3p showing a significant increase in expression after infection and inhibition of viral replication (Nersisyan et al., 2020b). Balmeh et al. reported miR-1307-3p as the miRNA with the highest affinity for the SARS-CoV-2 genome and plays an important role in the entry and spread of the virus in the cell (Balmeh et al., 2020).

### 3.2 Immune evasion

Although miRNAs have antiviral effects, some miRNAs can also evade the host’s antiviral response through other pathways to ensure their spread and achieve the effect of immune escape. SARS-CoV-2 creates an environment in which the virus can reproduce in host cells by affecting the expression levels of host genes (Barbu et al., 2020). It can be mainly divided into two types of mechanisms: interference of host miRNA and mutation of viral genome binding sites.

#### 3.2.1 Interfering with host miRNAs

Not only can host miRNAs have an effect on the SARS-CoV-2 genome, but some studies have shown that the SARS-CoV-2 genome can also have an effect on host miRNA and play a role in immune escape.

During infection, SARS-CoV-2 alters the expression patterns of host miRNAs. Studies suggest that SARS-CoV-2 can disrupt normal cellular homeostasis and weaken the body’s immune response by upregulating certain host mRNA levels controlled by host miRNAs and can also enhance its own virus replication ability by downregulating certain host miRNAs.

SARS-CoV-2 may act as a sponge to absorb miRNAs to reduce the host’s miRNA pool, promote the survival of infected cells, and ensure the continuity of their replication cycle. The bioinformatics study of Li et al. (2022) predicted that five human miRNAs (miR-374a-5p, let-7f-1-3p, miR-374a-3p, miR-548d-3p, and miR-23b-3p) would be absorbed by the SARS-CoV-2 genome as sponges. This study also suggests that the uptake of miR-302c-5p and miR-16-5p into the SARS-CoV-2 genome may lead to increased ACE2 expression and thus affect the severity of the viral infection. Furthermore, miR-16 binding sites are common in HCoV. MiR-16 can trigger apoptosis by downregulating the survival factor BCL2, and SARS-CoV-2 RNA can act as a sponge to sequester miR-16, thereby affecting the course of virus infection (Hum et al., 2021).

A computer-based study demonstrates that SARS-CoV-2 can alter miRNA network activity and alter cellular machinery to create a suitable environment for its replication and reproduction (Yousefi et al., 2020). Increasing ER membrane and ER folding capacity and blocking UPR-associated translation decay, inflammatory response, and apoptosis are some of the key mechanisms of virus survival. SARS-CoV-2 modulates host miRNAs involved in the UPR and/or inflammatory responses to fine-tune the ER of infected cells and protect itself from the immune system. Some of the miRNAs with more binding sites to the SARS-CoV-2 genome are UPR regulators (miR-34c-5p, miR-34a-5) or immune response regulators (miR-149-3p) (Bartoszewski et al., 2020). This study also found that SARS-CoV-2-mediated reduction of host miR-34a-3p and miR-495-5p levels increased XBP1s and BiP expression. Through miR-376b-3p, the virus may also regulate mTOR and autophagy pathways. In addition, some miRNAs also regulate immune responses, including miR-376a-3p, miR-99b-5p, miR-10a-5p, miR-376a-3p, miR-548av-5p, and miR-99b-5p (Bartoszewski et al., 2020).

Recent studies have found that the binding of specific target RNAs to miRNAs with broad complementarity can cause the degradation of the bound miRNAs. This pathway has been named TDMD, for Target RNA-Directed MicroRNA Degradation. Coronavirus RNA can cause TDMD (Fuchs Wightman et al., 2018). Nersisyan et al. computationally showed that miR-21-3p expression increased 8-fold after SARS-CoV infection, while miR-21-5p expression increased only 3-fold. They suggested that the reason for this difference might be that SARS-CoV-2 RNA triggered host miRNA degradation (Nersisyan et al., 2020b).

#### 3.2.2 Binding site mutations

RNA virus mutations are very frequent (Domingo et al., 2021). SARS-CoV-2 can alter the pathogenicity of the virus by mutating the binding site of the host miRNA (Hirabara et al., 2022). NSP is a class of transmembrane proteins in coronaviruses, and NSP3 is the largest protein encoded by the coronavirus genome and plays a critical role in replication and transcription (Lei et al., 2018). One study found that a potential miR-197-5p binding site was lost as a result of the Nsp3 synonymous C3037U mutation. MiR-197-5p was overexpressed in patients with cardiovascular disease, while this patient population exhibited increased susceptibility to SARS-CoV-2. In addition, the investigators predicted that three mutations within Nsp4 would affect miR-3935 and miR-18b-5p binding capacity (Hosseini Rad and McLellan, 2020). Another study found that the 28,881-3 GGG/AAC mutation in the nucleocapsid coding region caused changes in miRNA binding sites. If the patient is infected with the original virus containing the GGG sequence, the upregulation of miR-24-2-5p will have a protective effect on infection. If the patient is infected with the AAC mutant virus, miR-24-2-5p will no longer work and will be replaced by miR-299-5p targets this site, which may make cancer patients more susceptible to infection with the mutated virus. Besides, miR-3162-3p targeting the original subtype of SARS-CoV-2 may be one of the miRNAs that limit the spread of the original virus, but the loss of its targeting site in the mutant virus subtype may increase host resistance to the virus susceptibility to infection (Maitra et al., 2020).

### 3.3 Cytokine storm

The infection process of SARS-CoV-2 is mainly divided into two clinical phases, a viral infection of target cells and tissues and a state of severe inflammation (Pascarella et al., 2020). Inflammatory responses such as cytokine storms are caused by pro-inflammatory genes such as IL-6, IL-8, IL-1ß, NF-kB, and STAT3 (Hirano and Murakami, 2020). It is well known that miRNAs are deeply involved in the regulation of cytokine expression (Palanisamy et al., 2012). A large number of miRNAs can regulate the expression of IL-1β, IL-6, and IL-8, and studies have shown that there are a large number of miRNA binding sites in the 3′UTR of IL-1β, IL-6, and IL-8 mRNA (Chou et al., 2018).

Multiple miRNAs are thought to play pro-inflammatory roles in the immune factor storm of SARS-CoV-2. A study confirmed that miR-155 expression is significantly elevated in COVID-19 patients and demonstrates that elevated miR-155 leads to elevated levels of pro-inflammatory cytokines (Soni et al., 2020). Another bioinformatics study identified 10 miRNAs that may play a role in triggering cytokine storms, among which miR-155-5p is one of the 10 identified miRNAs. They may regulate the expression of multiple signaling pathways including IL-2, NF-κB, and MAPK, and trigger the inflammatory response in SARS-CoV-2 (Chow and Salmena, 2020). Following the same pathway we found that another study identified miR-8066, miR-5197-3p, miR-4778-3p, and miR-6864-5p sequences in the SARS-CoV-2 genome. These SARS-CoV-2-mediated miRNAs alterations may act as autocrine or paracrine agonists in host cells to trigger pro-inflammatory cytokines due to increased NF-κB activity (Hirabara et al., 2022). Another study found that miR-146a, miR-21, and miR-142 also trigger inflammation through the activation of MAPK and NF-κB signaling in SARS-CoV-2 patients (Tang et al., 2020). Continuing investigations along miRNAs found that multiple studies have suggested that downregulation of miR-21 and miR-142, which target pro-inflammatory genes, will promote multiple cascade reactions and the activation of JAK-STAT cell signaling pathways, which have the effect of pro-inflammatory and aggravating diseases (Bai and Bian, 2022; Panda et al., 2022).

In addition to this, several other mechanisms play a role in cytokine storms. A KEGG pathway analysis revealed the involvement of the miR-320 family (miR-320a, miR-320b, and miR-320c) in the regulation of the TGF-β signaling pathway and adhesion junctions, which may be related to pro-inflammation and could also be considered relevant to the severity of SARS-CoV-2 (Cavalli et al., 2021). In a study by Giuliani et al., both miR-320b and miR-483-3p/5p, which may both target glucose/lipid metabolism and IGF1 gene expression, were found to be upregulated in mortality cases compared to survivors. And the findings suggest that their serum levels were elevated by 20% and were associated with a threefold increased risk of death during hospitalization in SARS-CoV-2 patients (Giuliani et al., 2022). Besides, Wilson et al. found that severe disease was associated with higher levels of IL6, IL10, IL22, CXCL10, CCL2, and CCL20 and lower levels of miR-495-3p, miR-451a, and miR-29b-3p. Both the CCL20/IL6/IL10/miR-451a model and the 3miRNA/11CC model established in this study provide some clues to reveal the developmental pattern of SARS-CoV-2 (Wilson et al., 2022).

There are also alterations in the expression of miRNAs whose pro-inflammatory mechanisms are not fully understood that have been suggested in several studies to be associated with the development of uncontrolled inflammation in SARS-CoV-2 patients. One study demonstrated that dysregulation of miR-1207-5p during infection may be responsible for the uncontrolled inflammation that occurs in most severe SARS-CoV-2 cases (Bertolazzi et al., 2020). In contrast, miR-618 was shown to be more expressed in SARS-CoV-2 patients than in healthy controls in a study by Li et al. They suggested that the differential expression of miR-618 was associated with immune dysfunction (Li C. et al., 2020).

Except for the regulation of immune factors, in the SARS-CoV-2 cytokine storm, the binding of SARS-CoV-2 stinger protein to the ACE2 receptor, followed by ACE/Ang II/AT1R axis activation, causes IL-6/STAT hyperactivation of the NF-κB axis (Mahmudpour et al., 2020). Simultaneous activation of NF-κB and STAT-3 enhances the NF-κB activation mechanism (IL-6 amplifier) (Murakami and Hirano, 2012). Cytokine storm can be triggered by excessive IL-6 activation in lung tissue (Chi et al., 2020). A computer study showed that upregulation of six host miRNAs targeting ACE2 and TMPRSS2 (miR-32-5p, miR-98-3p, miR-214-3p, miR-421, miR-423-3p and miR-1246) may cause a severe inflammatory response associated with causing severe SARS-CoV-2 (Calderon-Dominguez et al., 2022).

Not only host miRNAs can cause cytokine storms, but also SARS-CoV-2 miRNAs can play the same role. SARS-CoV-2-derived miR-147-3p may enhance TMPRSS2 expression (Liu et al., 2021). And six SARS-CoV-2 miRNAs targeting immune genes (SARS-CoV-miR-1-5p, SARS- CoV-miR-2-5p, SARS-CoV-miR-3-5p, SARS-CoV-miR-4-5p, SARS-CoV-miR-5-5p, and SARS-CoV-miR-6-5p) were discovered Target immune-related genes that play important roles in immune pathways such as TNF and chemokine signaling pathways (Satyam et al., 2021).

## 4 Prospects and research directions of ncRNAs in prevention, diagnosis and treatment

**FIGURE 3:**
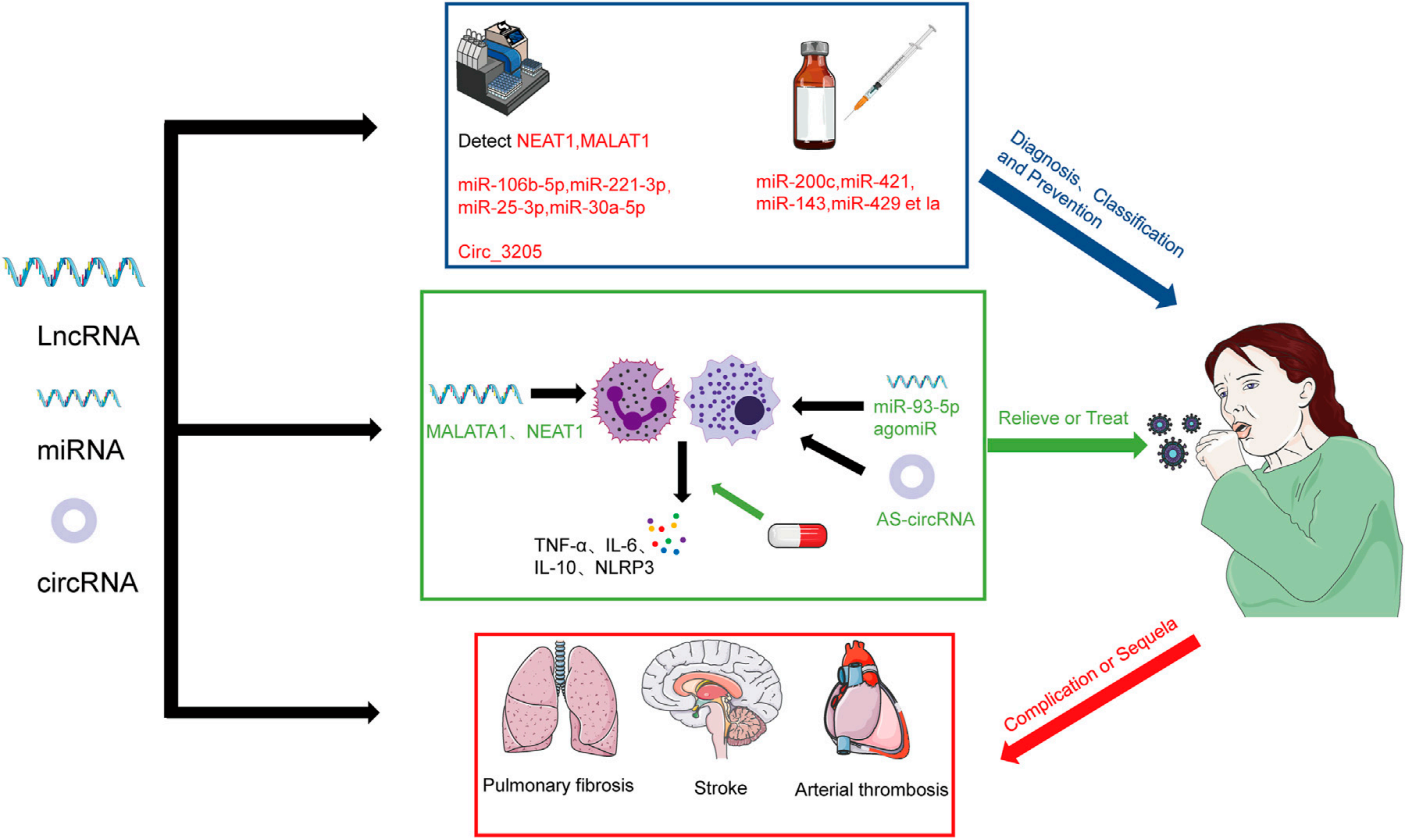
The prospect of the regulation of lncRNAs and miRNAs in SARS-CoV-2 infection. The severity of COVID-19 patients can be diagnosed and classified by detecting NEAT1, MALAT1, miR-106b-5p, miR-221-3p, miR-25-3p, miR-30a-5p, and Circ-3205 and prevent the infection of SARS-CoV-2 by regulating miR-200c, miR-421, miR-143, miR-429 et la. We also can relieve or treat by regulating MALAT1 and NEAT1 and using miR-93-5p agomiR and AS-circRNA. We also should concern about the role of ncRNAs in the complication or sequela of COVID-19.

It is even more critical that we learn more about the mechanisms by which the SARS-CoV-2 pandemic interacts with the human body as nations around the world start to relax their control strategies for SARS-CoV-2, which is progressively turning into a virus that lasts as long as influenza. We have described above the possible mechanisms of antiviral response, immune evasion, and cytokine storm of some ncRNAs in SARS-CoV-2 infection. Although many researchers have begun to study ncRNAs and viruses, and it has been confirmed that they play a non-negligible role in host-virus interaction (Damas et al., 2019), ncRNAs in SARS-CoV-2 infection remains relatively understudied in terms of the specific mechanisms. In order to improve our ability to use ncRNAs to assess whether patients are infected, forecast the severity of SARS-CoV-2 infection, and produce more efficient antiviral medications and vaccines, it is imperative that we continue to widely and completely study the mechanisms of interaction (Figure 3).

### 4.1 NcRNAs as diagnostic biomarkers

RT-PCR and SARS-CoV-2 antigen detection kits are now commonly used to detect the presence of SARS-CoV-2 infection (Jalandra et al., 2020). Rodrigues et al. found that lncRNA NEAT1 and MALAT1 were differentially expressed in saliva and nasopharyngeal swabs from SARS-CoV-2 positive and negative patients, with NEAT1 predominantly in saliva samples expression was upregulated and MALAT1 expression was upregulated in nasopharyngeal samples (Rodrigues et al., 2021). In a study by Martínez-Fleta et al. comparing the differential expression miRNAs in SARS-CoV-2 infection and community-acquired pneumonia, multivariate analysis displayed a combination of four miRNAs (miR-106-5p, miR-221-3p, miR-25-3p, and miR-30a-5p) that discriminated between both pathologies (Martínez-Fleta et al., 2022). Barbagallo et al. discovered that Circ-3205 was exclusively expressed in positive samples and that its level was strongly linked with the level of viral Spike mRNA, thus demonstrating the biomarker function of circRNAs (Barbagallo et al., 2022). NcRNAs appear to be used to detect some sequelae or complications in addition to the presence of infection. For instance, the study of Sabetian et al. demonstrated that lncRNAs can also be used as an early diagnostic biomarker for male infertility/reproductive disorders caused by SARS-CoV-2 (Sabetian et al., 2021), assisting patients in receiving treatment sooner.

Zhong et al. showed that the lncRNAs expression profile may identify between severe and non-severe COVID-19 cases, as well as between healthy people and infected patients (Zhong et al., 2022). Measurement of expression of the lncRNAs levels may help predict and monitor patient treatment, according to research by Badr et al. that found A2M-AS1 and NCBP2AS1-1 to be useful in differentiating between severe and moderate SARS-CoV-2 infections (Badr et al., 2022). Cheng et al. developed a 7-lncRNA panel with a good differential ability between severe and non-severe COVID-19 patients. It is evident that subtype iCluster2 and iCluster3 indicates more severe condition (Cheng et al., 2021). According to Meidert et al.’s discovery that 20 COVID-19 ARDS patients expressed miRNAs differently from COVID-19 pneumonia patients (Meidert et al., 2021), miRNA profiles may also be able to discriminate between severe and non-severe COVID-19 cases. In conclusion, ncRNAs can be employed in the clinic to diagnose SARS-CoV-2 infection, forecast its severity, and provide prompt and effective treatment approaches.

In contrast to conventional techniques of detection, the use of ncRNAs as a diagnostic biomarker can not only determine whether a person is infected with SARS-CoV-2 but also forecast the severity of the infection and the prognosis. The fact that several ncRNAs have been presented as biomarkers for diverse types of malignancies, however, raises doubt about their diagnostic specificity and may be a fatal defect. So it may be more practical to combine them with other clinical and biochemical markers, such as by adding the expression levels of some ncRNAs to the SARS-CoV-2 scoring system and risk classification.

### 4.2 NcRNAs as prevention targets

The scientific community currently recognizes vaccination as the most cost-effective method of preventing SARS-CoV-2 infection, but due to the mutation of SARS-CoV-2, numerous studies have found that the current vaccine against the original strain induces poor neutralization of antibodies with Omicron (Hoffmann et al., 2022; Zhang et al., 2022), leading to many cases of breakthrough infections. The goal of current research is to develop a new generation of vaccines or medications against Omicron, and Brevini et al. discovered that ursodeoxycholic acid (UDCA), a medicine with clinical approval, inhibited FXR signaling and decreased FXR activity, which reduced direct binding of FXR to the ACE2 promoter and ultimately downregulated ACE2 expression, which had the effect of suppressing SARS-CoV-2 infection (Brevini et al., 2023). This finding offers an innovative strategy for preventing SARS-CoV-2 infection. Given that miRNAs play a role in controlling the expression of ACE2 and TMPRSS2, using gene editing techniques to control the expression of miRNAs like miR-200c, miR-421, miR-143, and miR-429 may be able to suppress ACE2 and thereby prevent infection.

This preventive approach of suppressing ACE2 expression may have some disadvantages, such as the fact that FXR not only regulates ACE2 expression but also changes the expression of different groups of genes involved in BA homeostasis, lipid metabolism, and glucose balance. However, the use of gene editing of ncRNAs expression levels may be more targeted and less likely to produce additional side effects. In addition, long-term ACE2 deficiency may also result in the development of hypertension (Burrell et al., 2004). Therefore, this strategy for avoiding viral infection only be effective in the short term, and the application of reversible gene editing techniques may be able to overcome the problem.

### 4.3 NcRNAs as treatment targets

One of the key pathogenic mechanisms of SARS-CoV-2 infection is the detrimental influence of unregulated cytokines on the body, and inhibiting elements like IL-6 and NLRP3 inflammasomes may be a feasible treatment approach. As was already demonstrated, IL-6 and NLRP3 vesicles can be regulated by MALAT1 and NEAT1, and the use of IL-6 and NLRP3 blockers such as tolimumab and glibenclamide can lower morbidity and death (Paniri and Akhavan-Niaki, 2020). Gasparello et al. reported that the release of key proteins of the COVID-19 “cytokine storm” can be inhibited by mimicking the biological activity of miRNAs, and their study showed that transfection with miR-93-5p agomiR can downregulate SARS-CoV-2 S protein-induced IL-8 (Gasparello et al., 2021). According to Pfafenrot et al., these AS-circRNAs combined with short-circRNA could successfully target the 5′-UTP complex of SARS-CoV-2, limit the genomic expression of SARS-CoV-2, and 90% decrease viral replication in cell culture. Additionally, it was shown that functional antisense activity was invariably higher in the circRNAs backbone when comparing the generation of antisense sequences in circRNAs with that of linear RNA. As a result, AS-circRNA is a powerful and cutting-edge molecular strategy for regulating and targeting various RNAs (Pfafenrot et al., 2021). Direct regulation of ncRNAs by nanoparticles/CRISPR/EV may also be an effective therapy. However, due to the complexity of the ncRNAs interaction network and the fact that only a balanced and appropriate immune response facilitates the elimination of the virus, a thorough understanding of its particular action mechanisms network is essential.

### 4.4 Research directions of non-coding RNAs in SARS-CoV-2 infection

By 30 April 2023, at least one in ten people would have been infected with SARS-CoV-2 globally, according to WHO statistics. SARS-CoV-2 infection has been shown to have a variety of effects, including affecting respiratory, cardiovascular and hematological, neurological, and mental health, as well as the function of the endocrine and reproductive systems, which were previously thought to be transient in most SARS-CoV-2 infection sequelae in healthy young adults (Willi et al., 2021). According to another review, there are several potential late complications, including pulmonary fibrosis, atherothrombosis, stroke, and diabetes (Desai et al., 2022). The various complications and sequelae associated with SARS-CoV-2 infection significantly increase the burden on society, consume more medical resources, and hamper social advancement. Since we are likely to experience a continuous SARS-CoV-2 infection wave in the future, understanding the role of ncRNAs in this process is crucial.

Although the precise mechanisms causing COVID-19 long-term complications are still unknown, there is a growing amount of research suggesting that ncRNAs are involved. The presence of SARS-CoV-2 in cerebrospinal fluid has also been confirmed (Zhou et al., 2020). Approximately 40% of the lncRNAs discovered to date are specifically expressed in the brain (Harrow et al., 2012), and differential lncRNAs play an important role in neuronal, lung cancer, and various diseases (Wu et al., 2021). This evidence may show that lncRNAs play a role in COVID-19-induced neurological sequelae. Su et al. summarized the ncRNAs mechanisms in pulmonary fibrosis, such as overexpression of miR-142-5p and downregulation of miR-130a-3p expression leading to sustained pro-fibrotic effects of macrophages (Su et al., 2015). CircRNA TADA2A suppresses lung fibroblasts via miR-526b/Cav1 activation and lowers lung fibroblast proliferation via miR-203/CaV2 (Li J. et al., 2020).

Regular vaccination to maintain the potency and titer of neutralizing antibodies in our bodies has become an important method of preventing infection while we adjust to the wave of SARS-CoV-2 infection and accept the impact of its complications and sequelae. Tschumper et al. discovered that RNA-seq was able to distinguish specific expression profiles across the continuum of B cell differentiation into PCs, and confirmed that ncRNA plays a crucial role in B cell differentiation (Tschumper et al., 2022).

## 5 Conclusion

In conclusion, researchers are now concentrating on how ncRNAs regulate viral infections and the illness states. It has been confirmed that ncRNAs are engaged in viral-host interactions since a significant number of ncRNAs have been identified to be differently expressed in SARS-CoV-2 infection patients. The host activates and upregulates innate and adaptive immune responses to defend against SARS-CoV-2 invasion by regulating ncRNAs. On the other hand, SARS-CoV-2 interferes with host immune responses through hosts’ and self-encoded ncRNAs, promoting their own replication and leading to pathological damage to body tissues and organs. While there are likely still many ncRNAs to be discovered and their corresponding mechanisms need to be improved, the current discovery of ncRNAs involved in virus-organism interactions may only be the tip of the iceberg. Nevertheless, this knowledge offers new suggestions for the development of diagnostic targets and antiviral medications. Additionally, the research center has gradually shifted from the previous SARS-CoV-2 infection treatment to SARS-CoV-2 infection complications and sequelae, as SARS-CoV-2 has gradually entered our daily life. The role of ncRNAs in this also helps us to understand the pathogenesis of the corresponding complications and sequelae and serve as diagnostic biomarkers, prevention, and treatment targets.
